# Signal-independent timescale analysis (SITA) and its application for neural coding during reaching and walking

**DOI:** 10.3389/fncom.2014.00091

**Published:** 2014-08-19

**Authors:** Miriam Zacksenhouse, Mikhail A. Lebedev, Miguel A. L. Nicolelis

**Affiliations:** ^1^Brain-Computer Interfaces for Rehabilitation Laboratory, Department of Mechanical Engineering, Technion – IITHaifa, Israel; ^2^Department of Neurobiology, Center for Neuro-Engineering, Duke UniversityDurham, NC, USA

**Keywords:** timescales, rate modulation, doubly stochastic Poisson processes, rate coding, brain-machine interface, signal to noise ratio

## Abstract

What are the relevant timescales of neural encoding in the brain? This question is commonly investigated with respect to well-defined stimuli or actions. However, neurons often encode multiple signals, including hidden or internal, which are not experimentally controlled, and thus excluded from such analysis. Here we consider all rate modulations as the signal, and define the rate-modulations signal-to-noise ratio (RM-*SNR*) as the ratio between the variance of the rate and the variance of the neuronal noise. As the bin-width increases, RM-*SNR* increases while the update rate decreases. This tradeoff is captured by the ratio of RM-*SNR* to bin-width, and its variations with the bin-width reveal the timescales of neural activity. Theoretical analysis and simulations elucidate how the interactions between the recovery properties of the unit and the spectral content of the encoded signals shape this ratio and determine the timescales of neural coding. The resulting signal-independent timescale analysis (SITA) is applied to investigate timescales of neural activity recorded from the motor cortex of monkeys during: (i) reaching experiments with Brain-Machine Interface (BMI), and (ii) locomotion experiments at different speeds. Interestingly, the timescales during BMI experiments did not change significantly with the control mode or training. During locomotion, the analysis identified units whose timescale varied consistently with the experimentally controlled speed of walking, though the specific timescale reflected also the recovery properties of the unit. Thus, the proposed method, SITA, characterizes the timescales of neural encoding and how they are affected by the motor task, while accounting for all rate modulations.

## Introduction

The timescales of neural encoding is important for understanding neural processing and for successfully interpreting recorded neural activity (Shadlen and Newsome, 1994; de Ruyter van Steveninck et al., 1997; Borst and Theunissen, 1999; Jacobs et al., 2009). In the context of rate-coding, we focus on characterizing those timescales, independent of which signals modulate the rate. Those may include: (i) encoded signals under experimental control, (ii) encoded task-relevant signals that are not under experimental control and thus may vary from trial to trial, and (iii) task-irrelevant signals. The last two groups are referred to as “hidden” signals, and we are especially interested in the second group of hidden task-relevant signals. Those may include, for example, the estimated state or estimation error during reaching movements (Desmurget and Grafton, 2000; Wolpert and Ghahramani, 2000; Krigolson and Holroyd, 2006; Shadmehr and Krakauer, 2008).

The potential contribution of hidden signals to trial-to-trial variability in neuronal responses has been noted in the context of different tasks, including skilled reaching movements (Churchland et al., 2006a,b; Mandelblat-Cerf et al., 2009) motor adaptation (Mandelblat-Cerf et al., 2009) and decision making (Churchland et al., 2011). During skilled reaching movements, trial-to-trial variations in preparatory neural activity were predictive of variations in reach, and were interpreted to reflect variations in movement planning (Churchland et al., 2006a). During novel visuo-motor tasks, trial-to-trial variability in spiking activity increased in early adaptation stages before returning to initial levels at the end of learning, and was interpreted to reflect enhanced exploration or adjustments of the internal models (Mandelblat-Cerf et al., 2009). During decision making, trial-to-trial variations in the underlying rate were shown to increase until the decision was made, in agreement with the hypothesis that they encode trial-specific evidence accumulation (Churchland et al., 2011).

Ideally we should quantify and analyze the contributions of the first two groups of signals, i.e., only those that are task-relevant, whether under experimental control or hidden. However, this is hampered by the inability to control or measure hidden task-relevant signals. Current methods for analyzing neuronal timescales, which have focused mainly on sensory neurons, are based on averaging the response over repeated trials with the same stimuli (Borst and Theunissen, 1999; Warzech and Egelhaaf, 1999; Butts et al., 2007), or computing the mutual information between known stimuli and their reconstruction (Rieke et al., 1997; Dimitrov and Miller, 2000). In either case, the analysis accounts only for signals that are under experimental control, i.e., the first group of signals.

**Figure 1:**
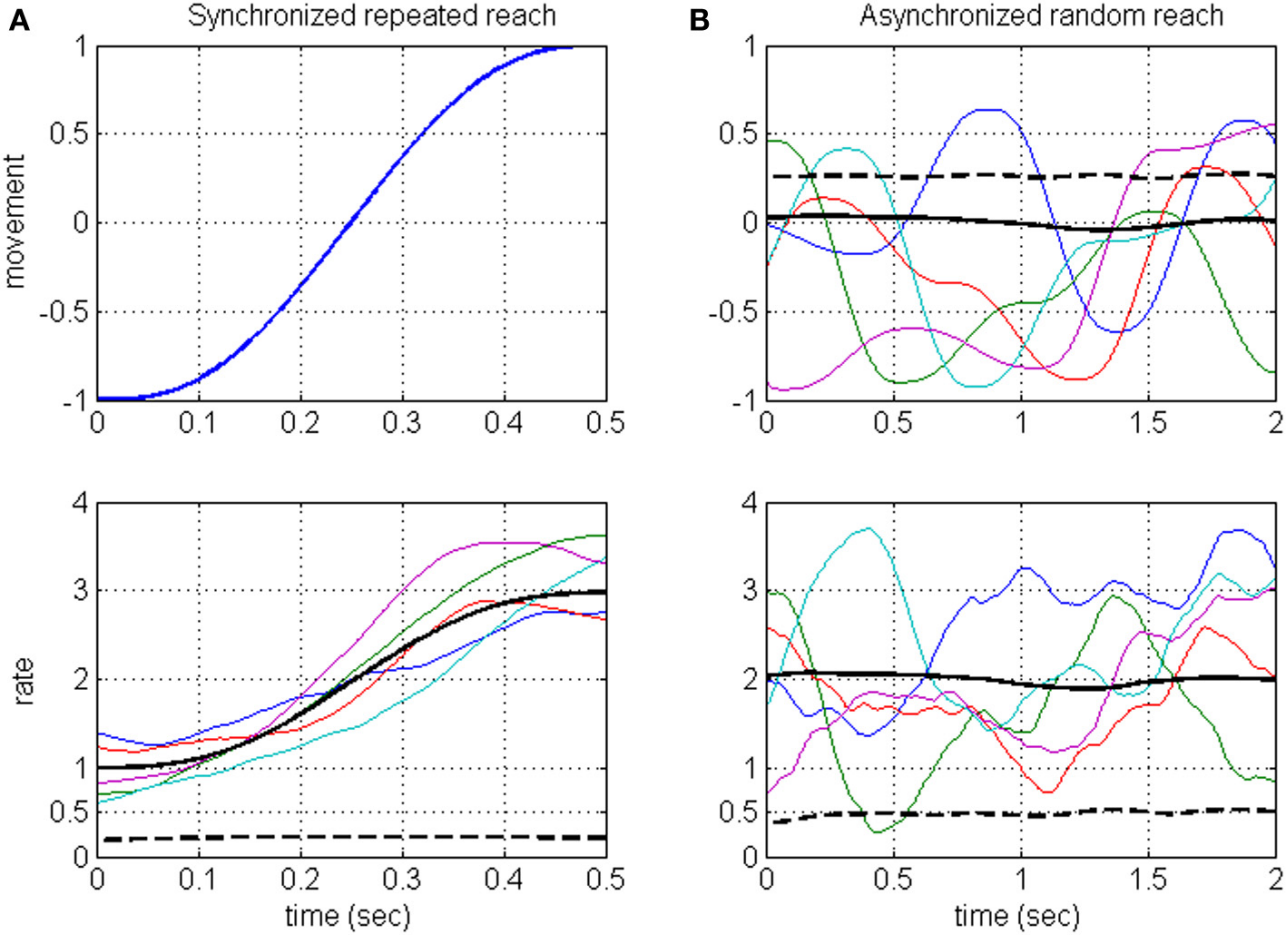
**Illustration of the averaging approach (A) vs. the proposed approach (B) when the rate encodes both the movement and a hidden signal**. Averaging methods are based on repeating the same movement (**A**, upper panel) and synchronizing the resulting neural activity and hence the rate (**A**, lower panel). Synchronized average (solid black) is considered the signal, while the variance of the hidden signal (dashed black) is considered noise. The proposed method considers asynchronous sequences of random reaching movements (**B**, upper panel) so the resulting rate (**B**, lower panel) is a stationary process whose variance (dashed black) captures the variance of both of the encoded signals [dashed black lines, **(A)** upper panel and **(B)** lower panel, respectively]. Each movement has a minimum jerk profile over 500 ms. Random reaching movements **(B)** are 2 s long sequences of reaching movements between random targets. Colored traces: representative samples. Black solid and dashed lines: ensemble mean and variance computed across an ensemble of 500 samples.

Neurons in the motor system are known to encode a large number of signals (Georgopoulos, 2000; Johnson et al., 2001; Scott, 2003), and are also expected to encode hidden signals, such as the estimated state and errors (Desmurget and Grafton, 2000; Wolpert and Ghahramani, 2000; Krigolson and Holroyd, 2006; Shadmehr and Krakauer, 2008). Timescale analysis that is based on synchronized averaging would miss the contribution of those signals that are not under experimental control, including hidden task-relevant signals. This is illustrated in Figure 1A (detailed in Section Rate-modulations Signal-to-noise ratio of DSPP), where the rate is assumed to encode both a movement related signal and a hidden signal: synchronized averaging captures the rate-encoded movement but not the rate-encoded hidden signal. Instead, we focus on analyzing the timescales associated with the underlying rate modulations, independent of which signals modulate the rate. The proposed method considers asynchronous sequences of random reaching movements (Figure 1B, upper panel) so the resulting rate (Figure 1B, lower panel) is a stationary process whose variance (dashed black) captures the variance of both of the encoded signals (dashed black lines, Figure 1A upper panel and Figure 1B lower panel, respectively). Thus, in contrast with standard averaging methods, the proposed method captures also the contribution of hidden signals. Admittedly, the hidden signals may include not only task-relevant (group 2) but also task-irrelevant (group 3) hidden signals. Hence, the method is best used in comparing the timescales across different task conditions. Here we demonstrate the power of this method by comparing the timescales in neural activity during different phases of experiments with brain-machine interfaces (BMIs) and during locomotion at different speeds.

Timescales are assessed under the sole assumption that spike trains are realizations of dead-time modified doubly stochastic Poisson processes (DSPP) (Snyder, 1975; Cox and Isham, 1980; Johnson, 1996; Gabbiani and Koch, 1998). DSPPs are the simplest point processes that can describe rate modulations by stochastic signals. This model is pertinent for describing spike trains recorded during reaching and walking when the rate might be modulated by a number of biologically relevant stochastic signals. These signals may include not only measurable variables, such as the velocity of movement, but also internal or hidden signals (e.g., internal state estimations; Wolpert and Ghahramani, 2000; Shadmehr and Krakauer, 2008), which cannot be measured or controlled directly (Zacksenhouse et al., 2007). The effect of dead-time is explicitly investigated to account for absolute refractory period. The effects of other deviations from the DSPP assumption are demonstrated via simulations.

Within the DSPP model, the modulated rate is considered as the signal conveyed by the unit. Only rate-conditioned spike count variations are considered noise (Churchland et al., 2011). Thus, we define the rate-modulations signal-to-noise ratio (RM-*SNR*) as the ratio between the variance of the rate and the variance of the Poisson noise (Section Rate-modulations Signal-to-noise ratio of DSPP). Theoretical analysis, presented in Section Bandwidth Effect and Appendix A, indicates that at short bin-widths RM-*SNR* should increase linearly with the bin-width, while at long bin-widths it should saturate. The analysis is extended in Section Dead-time Effect and Appendix B to include the effect of refractory period (dead-time modified DSPP). Section Simulations describes the simulations that are used to demonstrate the proposed methods. Section Timescales Analysis proposes to capture the trade-off between RM-*SNR* and update-rate by the ratio between RM-*SNR* and bin-width. The variations of this ratio with the bin-width are related to the interaction between the refractory properties of the unit and the spectral properties of the encoded signals, and the bin-widths at which this ratio peaks are related to the timescales of neural encoding. The effects of deviations from the DSPP assumption are evaluated by analyzing simulated realizations of doubly stochastic Gamma processes (DSGPs).

After demonstrating the analysis on simulated realizations of both DSPPs and doubly stochastic Gamma processes (DSGPs), it is applied to investigate the timescales of the neural activity recorded from cortical motor units during two experiments with Monkeys (described briefly in Section Experimental Methods). Sections SNR During BMI Experiments and Timescale During BMI Experiments present the analysis of spike-trains recorded during experiments with BMIs and demonstrate that the average timescale agrees well with the experimentally selected bin-width, though the timescales of individual units vary. The effect of the refractory period is assessed in Section Refractory Effects. Most importantly, Section BMI and Training Effects demonstrates that the timescales did not change significantly when switching to brain control or with training. During locomotion, the analysis identified the units whose timescales varied with the speed of walking—in agreement with the spectral analysis (Section Timescales During Walking). We conclude in Section Conclusions and Discussion, by discussing the proposed signal-independent timescale analysis (SITA) and its significance for investigating the timescales of neural-rate coding under different task conditions, and how they are related to the spectral content of the encoded signals and the refractory properties of the unit.

## Materials and methods

### Modeling and analysis methods

#### Rate-modulations Signal-to-noise ratio of DSPP

The Poisson process is the simplest point process in which the probability of an event—a spike in the case of neural activity, is assumed to be independent of the history of the spike train. Inhomogeneous Poisson processes are the simplest point processes that can describe rate modulations, i.e., the probability of a spike may change with time (Snyder, 1975; Cox and Isham, 1980; Johnson, 1996; Gabbiani and Koch, 1998). Here we consider DSPP, which is a subclass of inhomogeneous Poisson processes whose rate is modulated by stochastic signals (Snyder, 1975; Cox and Isham, 1980; Johnson, 1996; Gabbiani and Koch, 1998). Thus, the statistics of spike trains generated by DSPPs are determined by two factors: stochastic changes in instantaneous spike-rate and Poisson probability of spike occurrence.

Considering long intervals of movements, the stochastic signals that modulate the instantaneous rate, and thus the instantaneous rate itself, are assumed to be stationary. This is justified since we consider long sequences of movements (reaching or stepping) that are not synchronized to any event, and thus may start at an arbitrary phase of the movement. This approach is illustrated in Figure 1 and contrasted with synchronized averaging, using a simple example where the rate is assumed to encode both the position (along 1-dimension) during a reaching movement and an independent hidden signal. Figure 1A illustrates the averaging method, where the same reaching movement (upper panel) is repeated, and the resulting spike-rates (lower panel, depicting a sample of 5 realizations) are synchronized to movement initiation. The resulting synchronized rate is a *non-stationary* process with time-varying mean (solid black line, averaged over a simulated sample of 500 realizations). Figure 1B illustrates the proposed approach, showing a sample of five movement sequences (upper panel) starting at random phases (for illustration, each sequence includes four consecutive reaching movements between random targets). The resulting spike-rates (lower panel) are not synchronized and thus generate a *stationary* process with constant mean and variance (solid and dashed black lines, respectively, based on a sample of 500 realizations). The rate-variance (Figure 1B, lower panel) captures the contributions of both the movement (whose variance is depicted as black dashed line in Figure 1B, upper panel), and the hidden signal (whose variance is depicted as black dashed line in Figure 1A, lower panel).

Thus, the instantaneous spike-rate λ(*t*) = λ_0_ +$\tilde{\lambda }$ (*t*) is assumed to be a stationary stochastic process with mean rate λ_0_ and zero-mean rate modulations $\tilde{\lambda }$ (*t*). In realizations of DSPP, the distribution of spike-counts, *N*_*T*_, in bins of size *T* is determined by the integrated spike-rate during the bin:

(1) $$\Lambda_{T}(t)=\int_{t-T/2}^{t+T/2}\lambda (\sigma )d\sigma ,$$

and its statistics are given by (Snyder, 1975; Zacksenhouse et al., 2007):

(2) $$\begin{array}{l} \;E[N_{T}]=E[\Lambda_{T}]\\ Var[N_{T}]=Var[\Lambda_{T}]+E[\Lambda_{T}] \end{array}$$

The last equation can be interpreted as a decomposition of the total variance in the spike-counts into the variance of the modulated rate, *Var*[Λ_*T*_], and the variance of the noise *E*[Λ_*T*_] = *E*[*N*_*T*_] (Zacksenhouse et al., 2007). A similar decomposition was derived for general point processes from the total variance equation (Churchland et al., 2011), however the restriction to DSPPs assures that the meaning of the two sources of the neural variance is clearly defined (as further detailed in Section Conclusions and Discussion), and facilitates explicit analysis of the effect of the refractory period, as detailed in Section Dead-time Effect.

Considering the modulated rate as the signal, the RM-*SNR* is defined as:

(3) $$SNR_{T}\equiv \frac{Var[\Lambda_{T}]}{E[\Lambda_{T}]},$$

Using Equation (2), RM-*SNR* can be estimated from the mean and variance of the binned spike-counts as:

(4) $$S\hat{N}R_{N_{T}}=\frac{Var[N_{T}]-E[N_{T}]}{E[N_{T}]}=F-1$$

where the hat denotes estimation, and *F* is the Fano factor, defined as the ratio between the variance and the mean of the spike-counts (Dayan and Abbott, 2001). Thus, for the simple DSPP case, the RM-*SNR* is the deviation of the Fano-factor from its value for the homogeneous Poisson process, where *F* = 1. Positive RM-SNR reflects rate modulations and characterizes irregular spike trains (i.e., spike trains whose variance is larger than their mean and thus larger than the variance of a homogeneous Poisson process with the same mean). Expressing the *SNR* is advantageous for further analysis of the timescales, as detailed in Section Timescales Analysis.

#### Bandwidth effect

The relationship between RM-*SNR* and the spectrum *S*_$\tilde{\lambda }$_ (ω) of the instantaneous rate $\tilde{\lambda }$ (*t*) is analyzed in Appendix A. Equation (A1) shows that:

(5) $$SNR_{T}=\frac{1}{\lambda_{0}T}\frac{1}{2\pi }\int_{-\infty }^{\infty }{\left|G_{W_{T}}(\omega )\right|}^{2}S_{\tilde{\lambda }}(\omega )d\omega$$

where $\left|G_{W_{T}}(\omega )\right|=T\left(\frac{\sin(\omega T/2)}{\omega T/2}\right)$ is the Fourier transform of the rectangular window of duration *T* (Bendat and Peirsol, 2000). Furthermore, under the assumption that the spectrum of the instantaneous spike-rate *S*_$\tilde{\lambda }$_ (ω) is band limited in the range [−ω_max_, ω_max_], Appendix A derives two asymptotes for RM-*SNR* that hold at short and long bin-widths, respectively. For short bin-widths *T*, i.e., when ω_max_ «2π /*T*, Equation (A2) indicates that RM-*SNR* increases linearly with the bin-width. For long bin-widths *T*, i.e., when ω_max_ »2π /*T*, Equation (A3) indicates that RM-*SNR* saturates.

#### Dead-time effect

During absolute refractory period, or dead-time τ_*d*_, the instantaneous firing rate is zero regardless of the modulating signals. The effect of the dead-time on the estimated RM-*SNR* is detailed in Appendix B, and, assuming that the dead-time is short compared to the dynamics of the instantaneous rate, an exact expression for *S*$\hat{N}$*R*_*N*_*T*__ is derived (Equation B4).

To gain more insight into the effect of the dead-time, the estimated *S*$\hat{N}$*R*_*N*_*T*__ is approximated (Appendix B, Equation B9) by:

(6) $$S\hat{N}R_{N_{T}}(\tau_{d})≅\frac{SNR_{T}}{\left[1+\tau_{d}\lambda_{0}\right]}-\frac{\tau_{d}\lambda_{0}\left(2+\tau_{d}\lambda_{0}\right)}{{\left[1+\tau_{d}\lambda_{0}\right]}^{2}}$$

The approximation indicates that *S*$\hat{N}$*R*_*N*_*T*__ estimated from a realization of a dead-time modified DSPP is a scaled and downward shifted version of *SNR*_*T*_ associated with the original, dead-time free, rate. The scaling reflects the effect of the dead-time on the integrated rate (Equation B6), and the shift reflects the additional effect on the variance of the spike-counts (Equation B3).

#### Simulations

The analysis is demonstrated on simulated realizations of point processes with known characteristics. Specifically, the instantaneous rate was generated as a stationary stochastic process by: (a) passing a unit variance white Gaussian noise through a 2nd order Butterworth filter with known cut-off frequency *f*_*cut*_, (b) scaling the resulting signal to achieve the desired variance of the instantaneous rate, and (c) adding a constant mean rate λ_0_. Finally, simulated spike trains were generated from the resulting instantaneous rate using the inverse distribution function technique (Johnson, 1996). Spike trains were generated as realizations of DSPPs, doubly stochastic Gamma processes (DSGP; Fujiwara et al., 2009) with constant shape parameter κ, or dead-time modified DSPPs or DSGPs with fixed dead-time. In summary, the simulated spike trains were characterized by five parameters: bandwidth defined by the cut-off frequency *f*_*cut*_ of the low-pass filter, mean (λ_0_) and variance (*V*_λ_) of the instantaneous rate, dead-time τ_*d*_, and the shape parameter for DSGP (κ, κ = 1 for DSPP).

We note that for homogeneous Gamma processes with κ >1, the probability of firing after a spike is reduced, resulting in relative refractory period, and the variance of the spike-counts is lower than for the homogeneous Poisson process with the same mean. In contrast, for homogeneous Gamma processes with κ <1, the probability of firing after a spike is enhanced, and the variance of the spike counts is larger than for the homogeneous Poisson process with the same mean.

#### Timescales analysis

**Figure 2:**
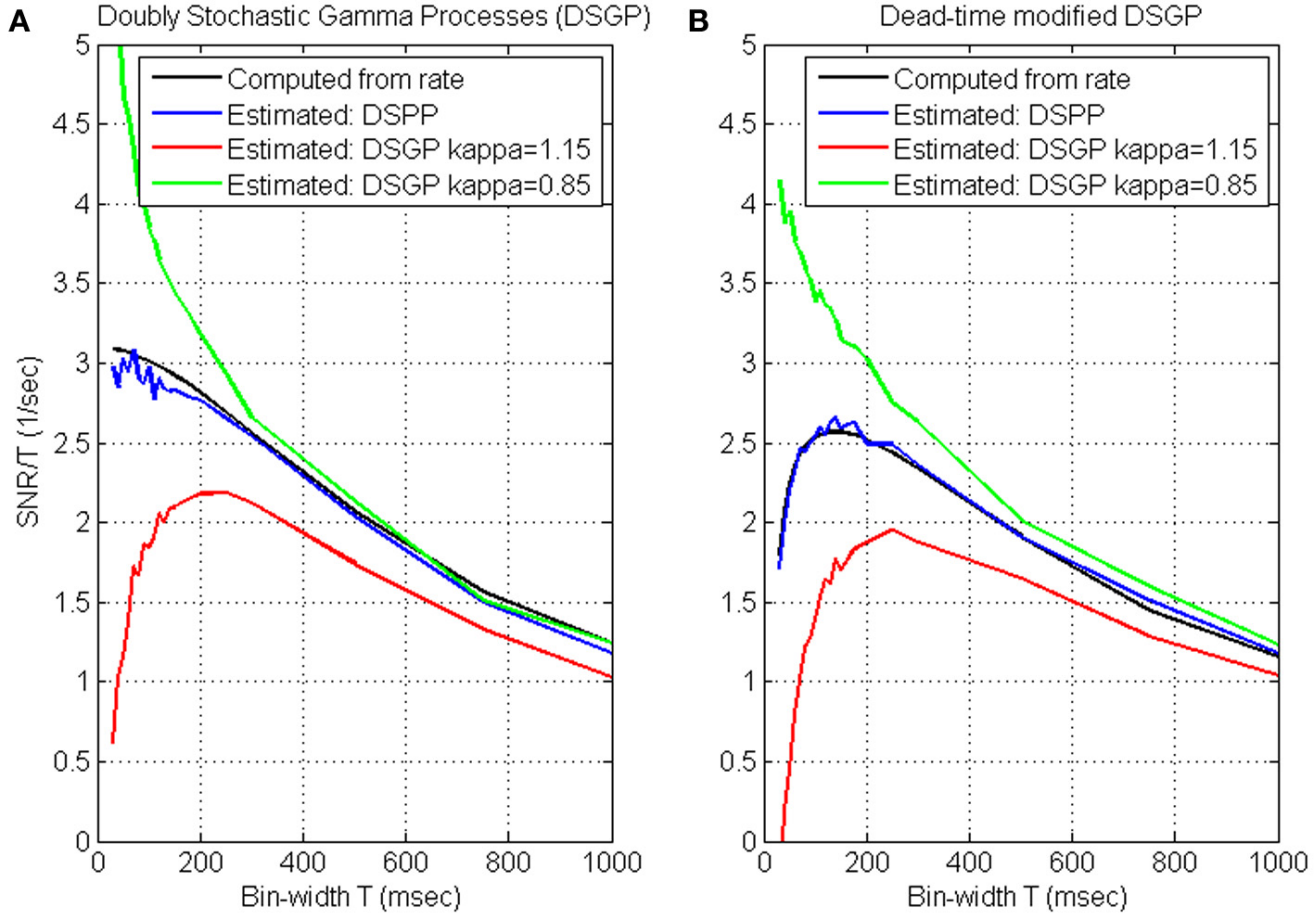
**Timescale analysis of simulated realizations of doubly stochastic Poisson and Gamma processes (DSPP, DSGP) (A) and dead-time modified (τ_*d*_ = 1) DSPP and DSGP (B)**. DSGPs are simulated with κ = 0.85, κ = 1 (i.e., DSPP), and κ = 1.15. All simulations were generated from the same instantaneous rate function generated with mean λ_0_ = 15 s^−1^, variance *V*_λ_ = 48 s^−2^, and cutoff frequency *f*_*cut*_ = 1.0 Hz. Estimated *S*$\hat{N}$*R*_*N*_*T*__/*T* (Equation 4) are compared with the values computed from the rate [using Equation (3) for the dead-time free DSPP in **(A)**, and Equation (B4) for the dead-time modified DSPP in **(B)**]. Analysis is conducted at bin-widths of 30, 40, 50, 60, 70, 80, 90, 100, 110, 120, 130, 140, 150, 175, 200, 250, 300, 500, 750, and 1000 ms.

While *SNR*_*T*_, and hence *S*$\hat{N}$*R*_*N*_*T*__, increase with the bin-width, the update rate decreases (Wu et al., 2006) and the reaction time increases (Cohen and Newsome, 2009). We quantify the trade-off between *S*$\hat{N}$*R*_*N*_*T*__ and update-rate by evaluating their product, i.e., the ratio *S*$\hat{N}$*R*_*N*_*T*__/*T*, as a function of the bin-width *T*, and characterize the timescale of neural encoding as the bin-width at which this ratio saturates or peaks. This is demonstrated in Figure 2, which depicts the variations of *S*$\hat{N}$*R*_*N*_*T*__/*T* with the bin-width for simulated realizations of DSPPs and DSGPs generated from the same realization of the instantaneous rate. The instantaneous rate was generated with mean λ_0_ = 15 s^−1^, variance *V*_λ_ = 48 s^−2^, and cut-off frequency *f*_*cut*_ = 1.0 Hz. The realizations analyzed in Figure 2A are dead-time free, while the realizations analyzed in Figure 2B include dead-time τ_*d*_ = 1 ms.

***Timescale analysis of DSPPs.*** The estimated *S*$\hat{N}$*R*_*N*_*T*__/*T* from both the dead-time free DSPP (Figure 2A, blue line) and dead-time modified DSPP (Figure 2B, blue line) closely match the values computed from the rate (Equations 3 and B4, respectively, black lines). The curves depict the expected effects of the spectral content and dead-time. At long bin-widths, *S*$\hat{N}$*R*_*T*_ should reach a constant asymptote (Equation A3), so *SNR*_*T*_/*T* should indeed decrease with the bin-width. The additive bias in estimating *S*$\hat{N}$*R*_*N*_*T*__ from dead-time modified DSPP is constant (Equation 6), independent of the bin-width, so, even when the spike-trains include a refractory period, the effect on the estimated *S*$\hat{N}$*R*_*N*_*T*__/*T* would diminish at long bin-widths, as is evident in Figure 2B. At the other extreme, i.e., when the bin-widths are short enough for the linear asymptote (Equation A2) to hold, *SNR*_*T*_/*T* should be approximately constant, in agreement with the estimated *S*$\hat{N}$*R*_*N*_*T*__/*T* for DSPP (blue curve, Figure 2A). Hence, the short bin-widths at which *S*$\hat{N}$*R*_*N*_*T*__/*T* saturates are indicative of the timescales above which the slower than linear increase in *SNR* does not justify the reduction in update rate.

Dead-time introduces a constant bias in estimating *S*$\hat{N}$*R*_*N*_*T*__ (Equation 6), which becomes increasingly significant as *SNR*_*T*_ diminishes at short bin-widths. Since the bias is negative, the estimated *S*$\hat{N}$*R*_*N*_*T*__/*T* becomes smaller as the bin-width becomes shorter, in agreement with the short bin-width trend of the blue curve in Figure 2B. The effect of the dead-time at short bin-widths limits the benefit of reducing the bin-width, and results in a peak in the *S*$\hat{N}$*R*_*N*_*T*__/*T* curve. The bin-width at which this peak occurs maximizes the trade-off between *S*$\hat{N}$*R*_*N*_*T*__ and update rate. We also note that below this bin-width the distortion due to the dead-time increases significantly.

***Timescale analysis of DSGPs.*** Applied to doubly stochastic Gamma processes (DSGPs) with scale parameter (κ) larger/smaller than 1, the estimated *S*$\hat{N}$*R*_*N*_*T*__/*T* under/over estimates the value computed from the rate, as demonstrated in Figure 2. In realizations of Gamma processes with κ >1, the occurrence of a spike has an inhibitory effect, resulting in relative refractory period. The estimated *S*$\hat{N}$*R*_*N*_*T*__/*T* curve in such a case (red line, Figure 2A) or when an absolute refractory period is also included (red line, Figure 2B) depict a peak similar to that for dead-time modified DSPP. As noted above (Section Simulations) realizations of homogeneous Gamma processes with κ >1 are characterized by spike-count variance that is smaller than the mean. The positive estimated *S*$\hat{N}$*R*_*N*_*T*__ (which implies variance larger than mean, see Equation 4) reflects the contribution of the variance of the stochastic rate.

In realizations of DSGPs with κ <1, the occurrence of a spike enhances, rather than inhibits, the probability of firing. The estimated *S*$\hat{N}$*R*_*N*_*T*__/*T* curve in such a case (green line, Figure 2A) increases sharply as the bin-width become shorter without saturating. For the specific parameters selected here, the excitatory recovery after a spike dominates the inhibitory effect due to the dead-time, and a similar increase is observed even with dead-time (Figure 2B).

***Timescales from *SNR*_*N*_*T*__/*T* curves.*** In summary, the analysis and simulations indicate that: (i) DSPPs are characterized by decreasing *S*$\hat{N}$*R*_*N*_*T*__/*T* curves that saturate at short bin-widths. In those cases, the bin-widths at which the *S*$\hat{N}$*R*_*N*_*T*__/*T* curve saturates are indicative of the timescales above which the slower than linear increase in *SNR* does not justify the reduction in update rate. (ii) dead-time modified DSPP or DSGP with relative refractory period (κ >1) are characterized by *S*$\hat{N}$*R*_*N*_*T*__/*T* curves that peak at some bin-width. The bin-width at which the *S*$\hat{N}$*R*_*N*_*T*__/*T* peaks maximizes the trade-off between *S*$\hat{N}$*R*_*N*_*T*__ and update rate, while limiting the distortion due to the refractory effect. (iii) DSPGs with excitatory recovery (κ <1) are characterized by decreasing *S*$\hat{N}$*R*_*N*_*T*__/*T* curves that remain convex even at short bin-widths without saturating. In those cases, the appropriate time-scale is not evident from the proposed analysis, since the higher *S*$\hat{N}$*R*_*N*_*T*__/*T* at short bin-widths reflect recovery effects rather than actual rate modulations. Further analysis is needed to select the bin-width that optimizes the trade-off between high *S*$\hat{N}$*R*_*N*_*T*__/*T* and small distortion due to the excitatory recovery effect.

**Figure 3:**
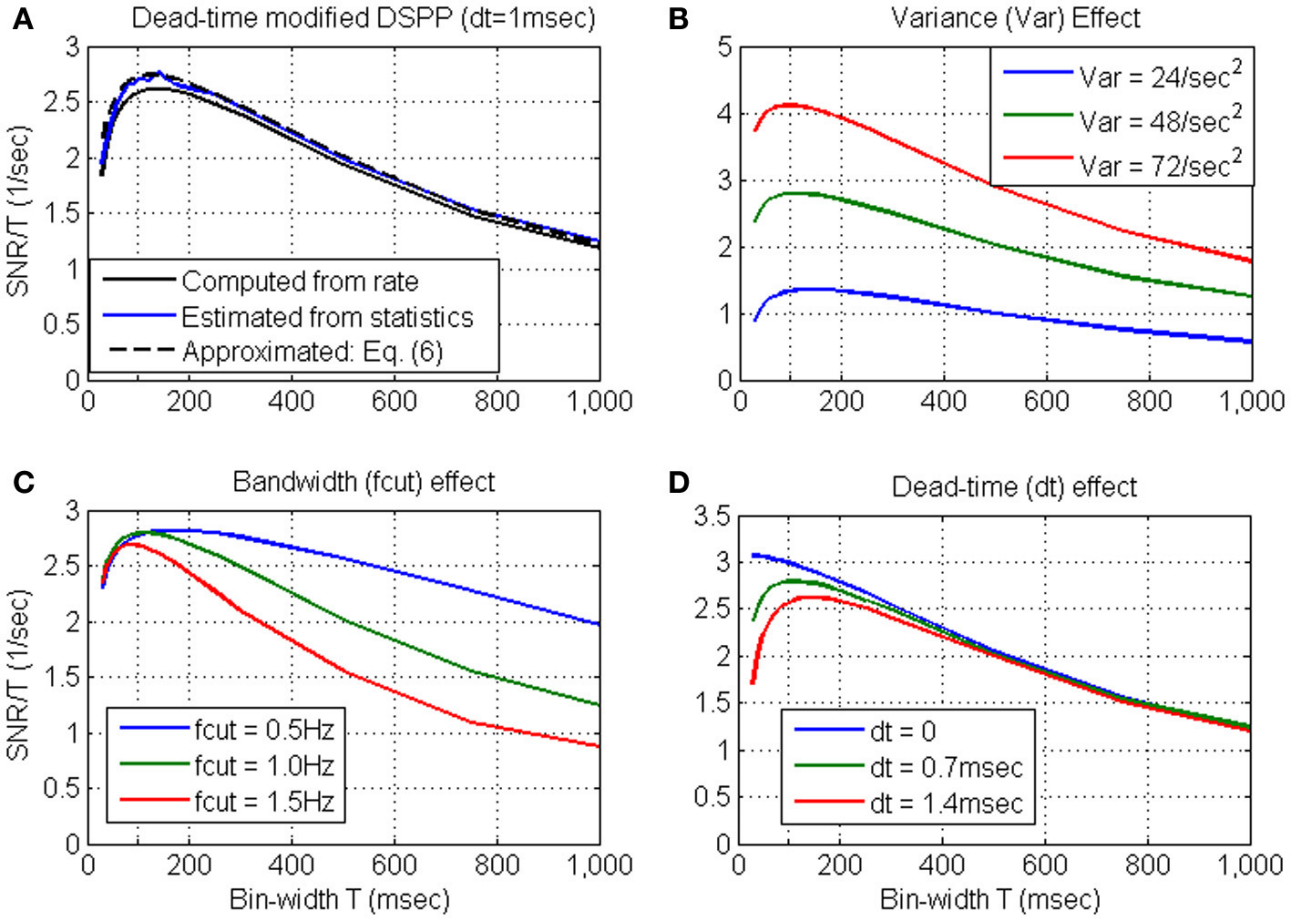
**Approximated *S*$\hat{N}$*R*_*N*_*T*__/*T* for dead-time modified DSPP (Equation 6) in comparison with the *S*$\hat{N}$*R*_*N*_*T*__/*T* computed from the rate (Equation B4) or estimated from the spike-counts (A)**. The approximation is used to demonstrate the effects of the variance **(B)** bandwidth **(C)** and dead-time **(D)**. Unless otherwise specified, the nominal parameters of the stochastic rate functions are: mean rate λ_0_ = 15 s^−1^, variance *V*_λ_ = 48 s^−2^, bandwidth *f*_*cut*_ = 1 Hz, and dead-time τ_*d*_ = 0.7 ms.

***Variance, bandwidth and dead-time effects.*** The effects of the variance, bandwidth and dead-time are further evaluated in Figure 3, based on the approximation derived in Equation 6. Figure 3A establishes that the approximated *S*$\hat{N}$*R*_*N*_*T*__/*T* (dashed black line) captures well the main effect of the dead-time. Hence, the above effects are evaluated by approximating *S*$\hat{N}$*R*_*N*_*T*__ directly from the simulated stochastic rate functions. Unless otherwise specified, the nominal parameters of the stochastic rate functions are: mean rate λ_0_ = 15 s^−1^, variance *V*_λ_ = 48 s^−2^, bandwidth *f*_*cut*_ = 1 Hz, and dead-time τ_*d*_ = 0.7 ms.

Figure 3B demonstrates that the major effect of increasing the variance (from *V*_λ_ = 24 s^−2^ to *V*_λ_ = 48 s^−2^ and *V*_λ_ = 72 s^−2^) is to increase the *SNR*. This is also accompanied by a shift in the peak location toward shorter bin-widths, due to the interaction between *SNR*_*T*_ and dead-time (Equation 6). Figure 3C demonstrates that as the cutoff frequency *f*_*cut*_ increases from 0.5 Hz to 1.0 Hz, and 1.5 Hz, *S*$\hat{N}$*R*_*N*_*T*__/*T* peaks at shorter bin-widths, capturing well the reduction in the relevant timescales. Figure 3D demonstrates that as the dead-time increases to 0.7 and 1.4 ms, *S*$\hat{N}$*R*_*N*_*T*__/*T* peaks at longer bin-widths. This trend reflects the increasing effect of the dead-time on the magnitude of the negative bias in Equation (6) (see note following Equation B9). Thus, for dead-time modified DSPP, the bin-width at which *S*$\hat{N}$*R*_*N*_*T*__/*T* peaks reflects the conflicting effects of the bandwidth of the encoded signals and the refractory period.

### Experimental methods

The analysis tools developed above to estimate the RM-*SNR* of spike-trains and evaluate their timescales are applied to spike-trains recorded form cortical units in two experiments with Monkeys: (a) BMI experiments and (b) Locomotion experiments.

#### Ethics statement

All experiments were conducted with approved protocols from the Duke University Institutional Animal Care and Use Committee and were in accordance with the NRC/NIH guidelines for the Care and Use of Laboratory Animals.

#### BMI experiments

As detailed in Carmena et al. (2003), the BMI experiments were conducted with macaque monkeys and included three control modes: (i) pole control, (ii) brain control with hand movements (BCWH), and (iii) brain control without hand movements (BCWOH). During pole control the monkey controlled the cursor on the screen using a hand held pole. Data from pole control was used to train a linear filter to predict the velocity from the neural activity. During brain control the cursor was controlled to follow the velocity predicted from the recorded neural activity using the previously trained linear filter. Initially, the monkey continued to move its hand during brain control (BCWH), but starting at the 7th session, the monkey stopped moving the hand (BCWOH). Spike trains were recorded from single and multi-units (see Section Refractory Effects for more details) in the primary motor area (M1), the pre-motor area (PMd), the supplementary motor area (SMA), and somato-sensory area (S1). Here we focus on significantly modulated units (*n* = 163), i.e., those units for which the null hypothesis that their ISI distribution was generated from a homogeneous Poisson process can be rejected at α = 0.05 significance level (Zacksenhouse et al., 2007).

#### Locomotion experiments

During the locomotion experiments, rhesus macaques either stood or walked bipedally on a treadmill in three different speeds, 12.5, 25, and 50 cm/s, as detailed in Fitzsimmons et al. (2009). Spike trains were recorded from single and multi-units (66 and 34%, respectively) in regions associated with the representation of the lower limbs in the primary motor (M1) and somatosensory (S1) cortices.

## Results

### SNR during BMI experiments

**Figure 4:**
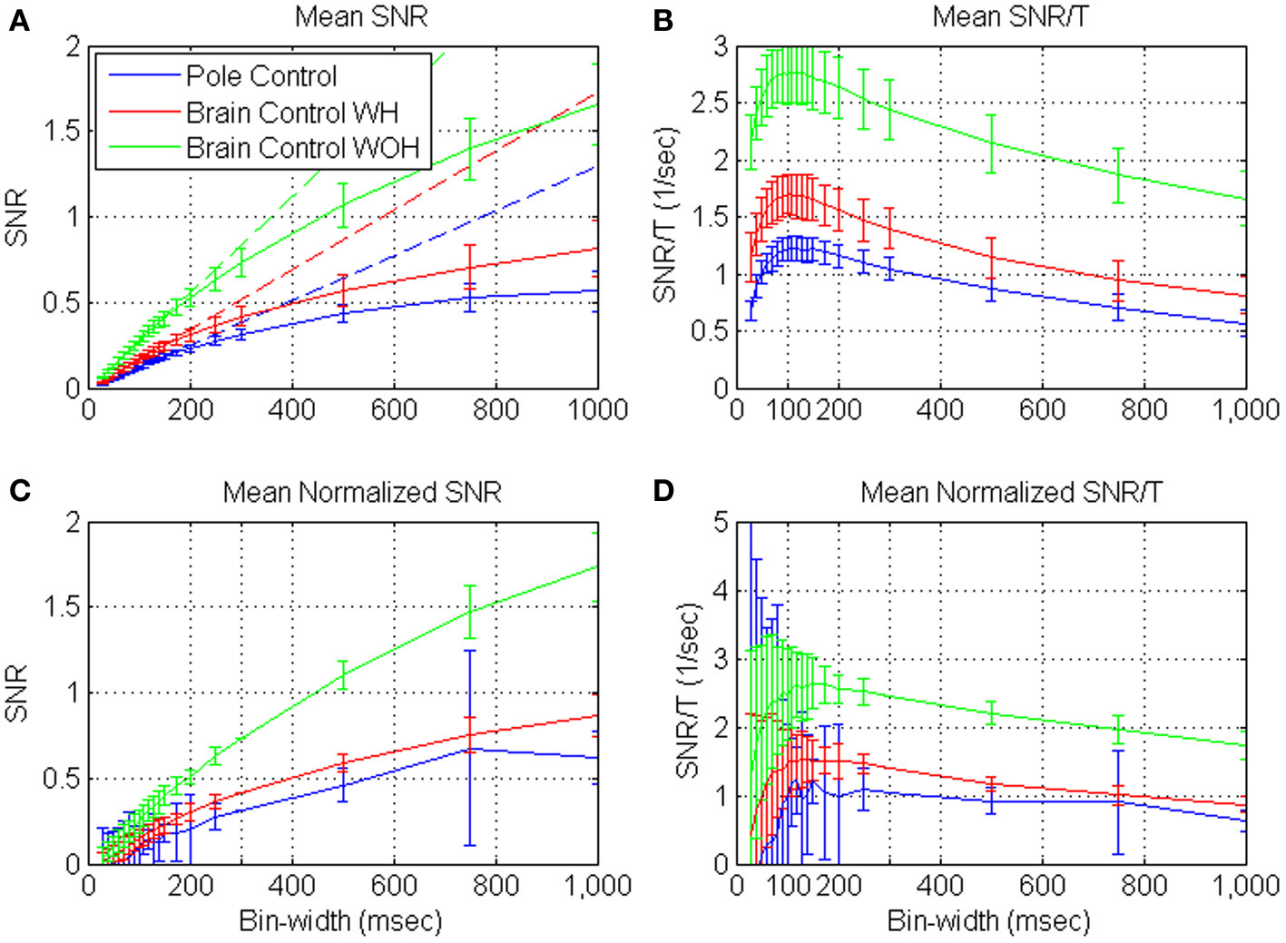
**Mean estimated *S*$\hat{N}$*R*_*N*_*T*__ (A) and normalized *S*$\hat{N}$*R*_*N*_*T*__ (C), and the corresponding *S*$\hat{N}$*R*_*N*_*T*__/*T* (B,D) of neural activity recorded from significantly modulated units (*n* = 163) during BMI experiments**. BMI experiments included pole control, brain control with hand movements (WH), and without hand movements (WOH). *S*$\hat{N}$*R*_*N*_*T*__ was computed from the spike-train of each unit in non-overlapping windows of 2-min. **(A,B)** are based on first averaging over the units at each window, and then computing the mean and standard deviation over 10 consecutive windows in each of the experimental mode. Hence, error-bars in **(A,B)** mark the standard deviations across 10 consecutive windows. **(C,D)** are based on first averaging the *S*$\hat{N}$*R*_*N*_*T*__ of each unit across all windows, and then scaling it so the normalized *S*$\hat{N}$*R*_*N*_*T*__ at bin-width of 300 ms is the same for all units and equal the original ensemble mean. Ensemble mean and standard deviation of the normalized *S*$\hat{N}$*R*_*N*_*T*__ were computed across units. Hence, error bars in **(C,D)** mark the standard deviations across the ensemble of units (resulting in zero standard deviation at 300 ms, which was used for normalizations). Dashed lines in **(A)** depict the linear fits for short bin-widths, over the range 50–150 ms.

Figures 4A,C depict the mean *S*$\hat{N}$*R*_*N*_*T*__ and mean normalized *S*$\hat{N}$*R*_*N*_*T*__ estimated from spike-trains of significantly modulated cortical units (*n* = 163) recorded during a BMI experiment, as a function of the bin-width *T*. Assuming ergodicity, Equation (4) was used to estimate the *S*$\hat{N}$*R*_*N*_*T*__ of each unit from its spike-counts statistics in non-overlapping windows of 2 min. The mean *S*$\hat{N}$*R*_*N*_*T*__ in Figure 4A was derived by first computing ensemble mean (over *n* = 163 units) at each window, and then computing the mean and standard deviation over 10 consecutive windows in each of the experimental modes (pole control and brain control with and without hand movements). Thus, error bars in Figure 4A depict the standard deviations of the ensemble-means over the 10 windows of time. Variations across the ensemble of units are described in Figure 4C, which depicts the ensemble mean and standard deviations of the normalized *S*$\hat{N}$*R*_*N*_*T*__. The normalized *S*$\hat{N}$*R*_*N*_*T*__ for each unit was computed by first averaging across all windows, and then normalizing by a scaling factor. The scaling factor was selected so the normalized *S*$\hat{N}$*R*_*N*_*T*__ at bin-width of 300 ms is the same for all units and equals the original ensemble mean. Thus, error bars in Figure 4C depict the standard deviations of the normalized *S*$\hat{N}$*R*_*N*_*T*__ across the ensemble of significantly modulated units (resulting in zero standard deviation at 300 ms, which was used for normalizations).

Figures 4A,C demonstrate that the mean estimated *S*$\hat{N}$*R*_*N*_*T*__ increases approximately linearly at short bin-widths and approaches a constant level at long bin-widths, in agreement with the expected asymptotes (Equations A2 and A3). Another striking feature is that at each bin-width, the mean *S*$\hat{N}$*R*_*N*_*T*__ in brain control is always higher than that in pole control; and is highest in brain control without hand movements. This extends our previous results (Zacksenhouse et al., 2007) for *T* = 100 ms—the bin-width used for operating the BMI.

### Timescale during BMI experiments

Figures 4B,D demonstrate that the mean *S*$\hat{N}$*R*_*N*_*T*__/*T* and mean normalized *S*$\hat{N}$*R*_*N*_*T*__ /*T* increase at short bin-widths, reach a wide peak around 100 ms, and decrease at long bin-widths. This pattern agrees well with the analysis and approximation of the *S*$\hat{N}$*R*_*N*_*T*__/*T* of dead-time modified DSPP, and with estimations derived from the simulated spike-trains of dead-time modified DSPPs (or DSGPs with κ <1, with or without dead-time) depicted in Figure 2.

**Figure 5:**
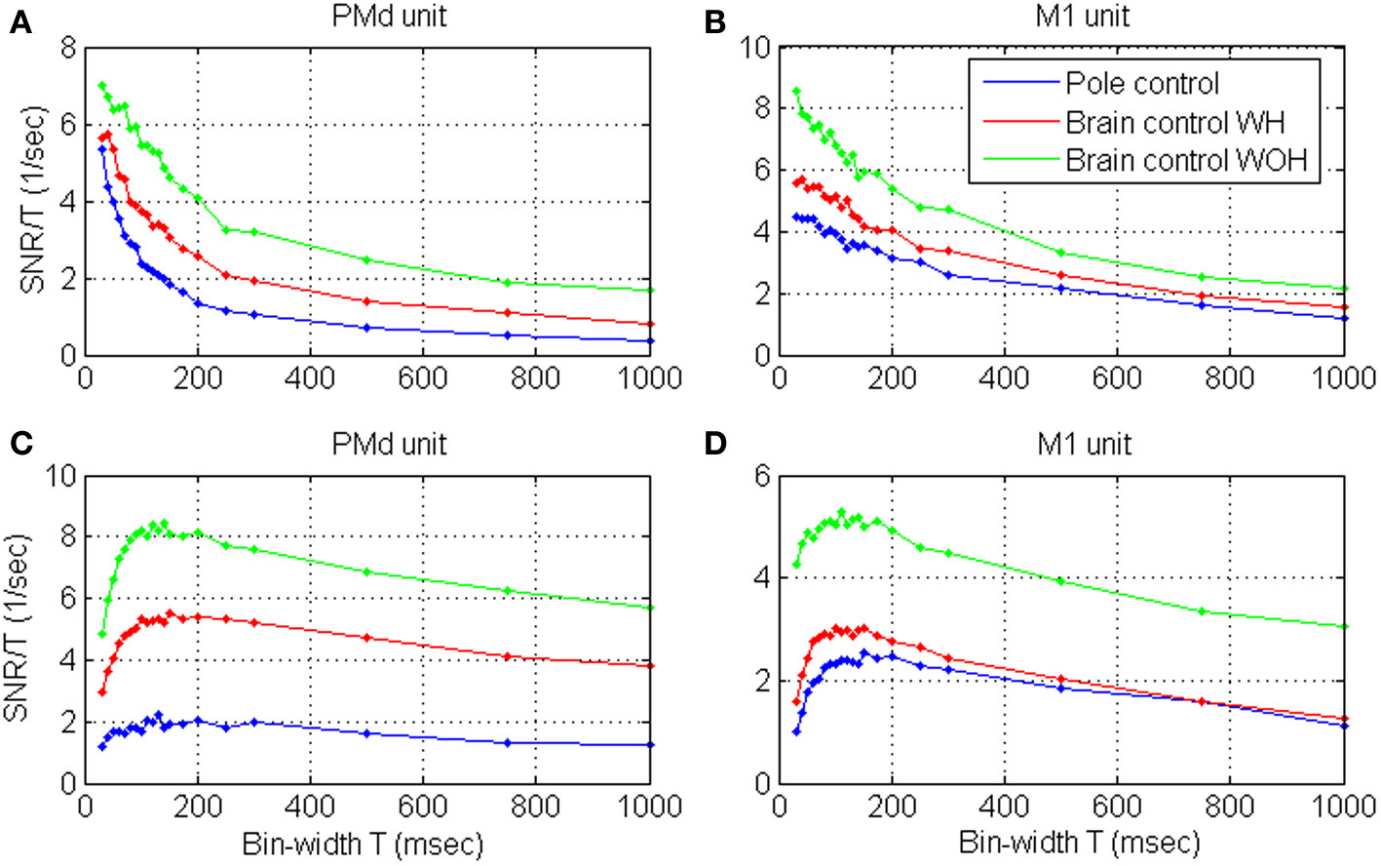
**Estimated *S*$\hat{N}$*R*_*N*_*T*__ /*T* as a function of the bin-width for four representative cortical units in PMd (A,C) and M1 (B,D), during pole control, and brain control with and without hand movements, WH and WOH, respectively**.

Focusing on individual units from PMd and M1, the *S*$\hat{N}$*R*_*N*_*T*__ /*T* curves shown in Figure 5 demonstrate two typical patterns: (i) curves with a wide peak around 100 ms (Figures 5C,D), similar to the average curves in Figures 4B,D and to the curves for dead-time modified DSPP or DSGP with κ <1, in Figure 2, or (ii) decreasing curves (Figures 5A,B), similar to the curves for DSPP or DSGP with κ >1 in Figure 2. The second type could be further divided depending on whether the curve saturates at short bin-widths as for DSPPs, or remains convex as for DSGPs with κ >1.

Units were classified as exhibiting a wide peak around 100 ms if the peak *S*$\hat{N}$*R*_*N*_*T*__/*T* was in the range of 50–150 ms, and as exhibiting decreasing *S*$\hat{N}$*R*_*N*_*T*__/*T* if the peak was below 50 ms. Note that *S*$\hat{N}$*R*_*N*_*T*__/*T* of few units peaked at bin-widths longer than 150 ms, and were not included in either group. To assure proper peak-detection, we restricted this classification and further analysis of the timescales to the significantly modulated units whose maximum *S*$\hat{N}$*R*_*N*_*T*__/*T* during pole control was larger than 0.5 s^−1^ (*n* = 109). Of those units, 59.7, 64.2, and 61.5% exhibited a wide peak around 100 ms during pole control, BCWH and BCWOH, respectively, while 25.7, 21.1, and 22.0% exhibited decreasing curves, respectively. The second group was further divided depending on whether the decreasing curve was convex at short bin-widths, i.e., whether *u*_2_ < (*u*_1_ + *u*_3_)/2 where *u*_1_, *u*_2_, *u*_3_ are the mean *S*$\hat{N}$*R*_*N*_*T*__/*T* in the bin-width intervals 30–50, 60–80, and 90–100 ms, respectively. About 15% of the curves were convex at short bin-widths (17.4, 11.0, and 15.6%, in pole control BCWH and BCWOH, respectively). In those cases, the time-scale analysis is not conclusive, and is either not appropriate since the recovery function is dominated by excitatory effects (as clarified in Section Timescales Analysis) or should be extended to shorter bin-widths.

The *S*$\hat{N}$*R*_*N*_*T*__/*T* estimated form spike-trains recorded during BMI experiments (Figures 4B,D, 5) vary with the bin-width in a similar way to the *S*$\hat{N}$*R*_*N*_*T*__/*T* of simulated spike-trains (Figure 2). Decreasing curves, which saturate at short bin-widths (Figure 5B, pole and BCWH) agree well with the assumption that the spike-trains are realizations of DSPP while decreasing curves that remain convex at short bin-widths (Figure 5A, pole and BCWH) agree well with the assumption that the spike-trains are realizations of DSGP with κ <1. Curves that peak (Figures 5C,D) agree well with the assumption that the spike trains are realizations of dead-time modified DSPP or DSGP with κ >1 (with or without dead-time), which are characterized by relative, rather than absolute, refractory period.

#### Refractory effects

To assess the potential contribution of refractory effects, we estimated the refractory interval from the ISI distribution. The minimum ISI in all units was 0.675 ms, and most units (95% of the significantly modulated units) had minimum ISI of less than 1 ms. Since a single ISI is prone to measurement errors, we estimated the dead-time as the interval which was exceeded by 99% of the ISIs in at least one mode of operation. Of the 109 units described above, 50% had dead-time greater than 1.6 ms, and thus could be considered single-units (Hatsopoulos et al., 2004). The percent of units having decreasing curves was similar, independent of whether the dead-time was short or long (below or above 1.6 ms); but the percent of units having time-scales longer than 150 ms was larger for units with long dead-time (22.4% on average, across all modes, compared with 8% for units with short refractory interval). Furthermore, the bin-widths at which *S*$\hat{N}$*R*_*N*_*T*__ /*T* peaked had a moderate positive correlation with the product of the dead-time and the mean rate (coefficient of correlation of 0.4, 0.42, 0.48 in pole control BCWH and BCWOH, respectively). Both the positive correlation and its moderate value are in agreement with Equation (6), which indicates that this product reduces the *S*$\hat{N}$*R*_*N*_*T*__ (and hence shifts the peak of the *S*$\hat{N}$*R*_*N*_*T*__ /*T* toward longer bin-widths, as clarified in Section Timescales Analysis), but that *S*$\hat{N}$*R*_*N*_*T*__ is also affected by other variables (i.e., *SNR*_*T*_).

### BMI and training effects

**Figure 6:**
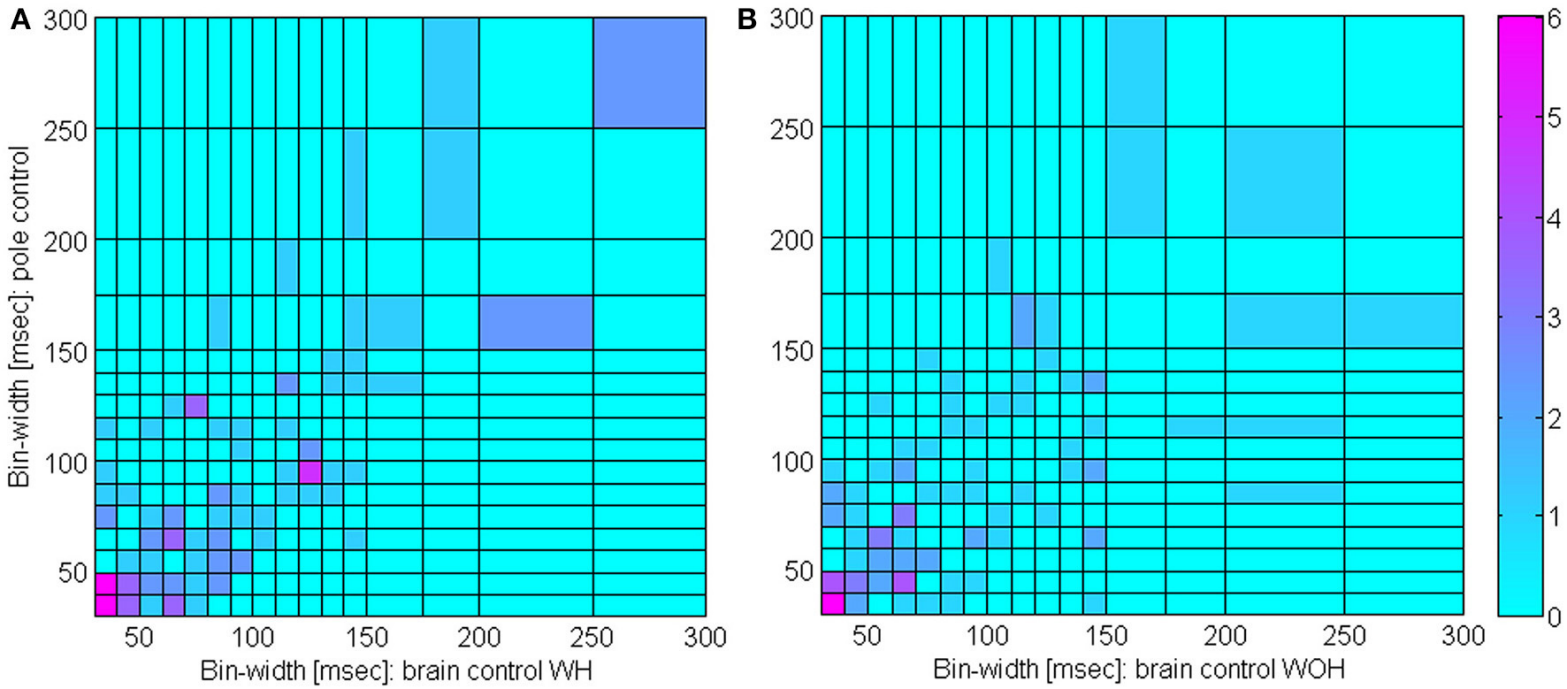
**Distribution of timescales during BMI experiments**. Comparison of bin-widths at which *S*$\hat{N}$*R*_*N*_*T*__/*T* curves peak during pole control and during either brain control with hand movements, (WH, **A**) or brain control without hand movements (WOH, **B**). Only significantly modulated units whose peak *S*$\hat{N}$*R*_*N*_*T*__/*T* during pole control was larger than 0.5 s^−1^ were included (*n* = 109).

Figure 5 indicates that the bin-width at which the *S*$\hat{N}$*R*_*N*_*T*__/*T* peaks remains approximately the same even after switching to brain control. Specifically, the *S*$\hat{N}$*R*_*N*_*T*__/*T* curves in pole control, BCWH and BCWOH peak at: 30, 40, and 30 ms for the PMd unit in panel **(A)**, 130, 150, and 140 ms for the PMd unit in panel **(B)**; 30, 40, and 30 ms, for the M1 unit in panel **(C)**; and 150, 150, and 110 ms for the M1 unit in panel **(D)**. The distribution of the bin-widths at which the estimated *S*$\hat{N}$*R*_*N*_*T*__/*T* peak and how they change when switching to brain control is summarized in Figure 6. The bin-width at which the estimated *S*$\hat{N}$*R*_*N*_*T*__/*T* peaks during pole control is similar to (within 50 ms of) the bin-width at which it peaks during brain control for most of the units (80.7 and 67.9% for BCWH and BCWOH, respectively).

**Figure 7:**
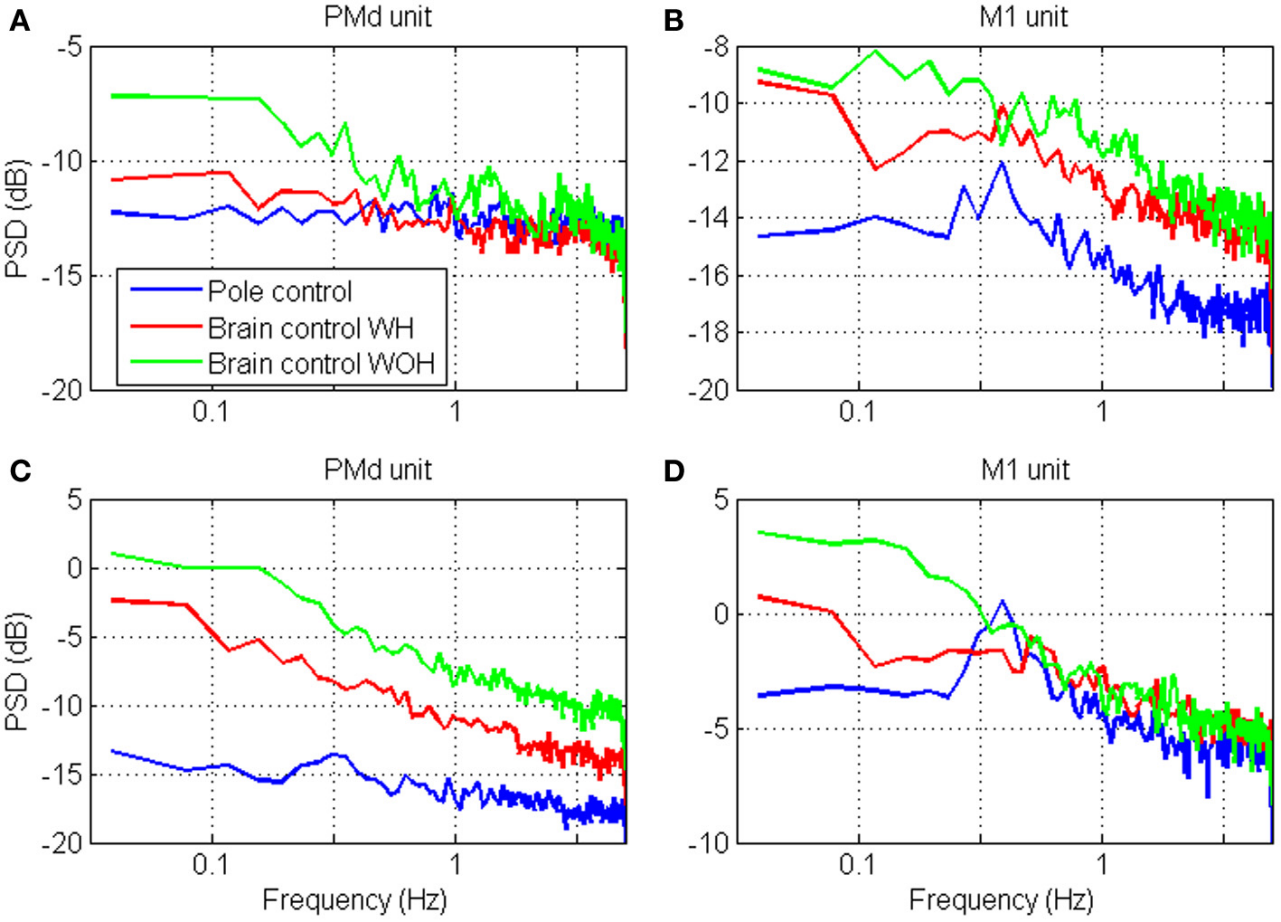
**Power spectrum density (PSD) of the neural activity analyzed in Figure 4**. The spectrums were computed from spike-counts in 100 ms bins, with frequency resolution of 0.039 Hz.

The spectral content of the spike-counts (in 100 ms bins) of the four units whose timescales were analyzed in Figure 5, are depicted in Figure 7. Specifically, the power spectrum density (PSD) was computed from the spike-counts in bins of 100 ms after subtracting the mean and expressed in dB. The main effect of the transition to brain control is the increase in power, which is higher in brain control than in pole control and is highest in brain control without hand movements. This is consistent with the higher RM-*SNR* in brain control compared to pole control. The overall bandwidth of the activity of each unit remains relatively similar across the different BMI control modes [even when, as in panel **(D)**, the PSD of the activity during pole control portrays a small peak which is surpassed by the higher overall power during brain control]—consistent with the relatively similar shapes and peak locations of the different *S*$\hat{N}$*R*_*N*_*T*__/*T* curves for each unit in Figure 5.

**Figure 8:**
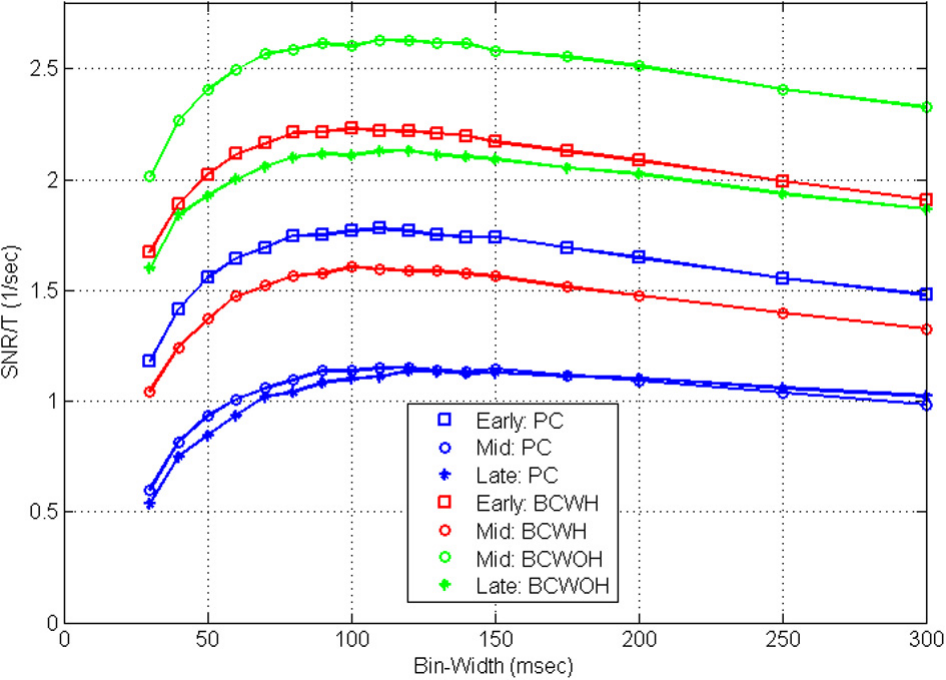
**Mean estimated *S*$\hat{N}$*R*_*N*_*T*__/*T* across the population of significantly modulated cortical units recorded in three different BMI sessions during pole control (PC), and brain control with and without hand movements (BCWH and BCWOH, repectively) as a function of the bin-width**. The three sessions include: an early session (3rd day); a mid session (7th day), and a late session (10th day). The 7th day is the first one in which BCWOH was performed, and in subsequent sessions BCWH was not perfomrned.

Figure 8 demonstrates that the shape of the *S*$\hat{N}$*R*_*N*_*T*__/*T* curves is also invariant to training. At later sessions, as training progresses, the *SNR* at each control mode and bin-width decreases (as detailed in Zacksenhouse et al., 2007) for bin-width of 100 ms). However, the effect of bin-width is similar and the *S*$\hat{N}$*R*_*N*_*T*__/*T* curves peak at a similar bin-width in all sessions.

### Timescales during walking

**Figure 9:**
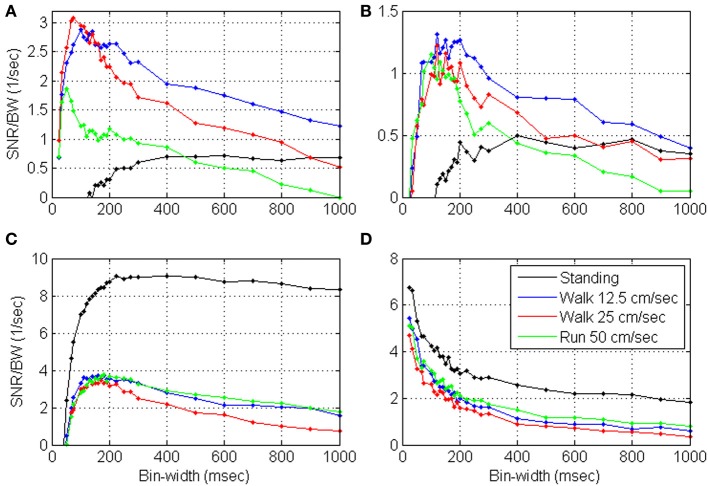
**Estimated *S*$\hat{N}$*R*_*N*_*T*__/*T* as a function of the bin-width for four representative cortical units (A–D) in the motor leg area while the monkey is standing (black) or walking at increasing speeds (12.5, 25, and 50 cm/s)**.

**Figure 10:**
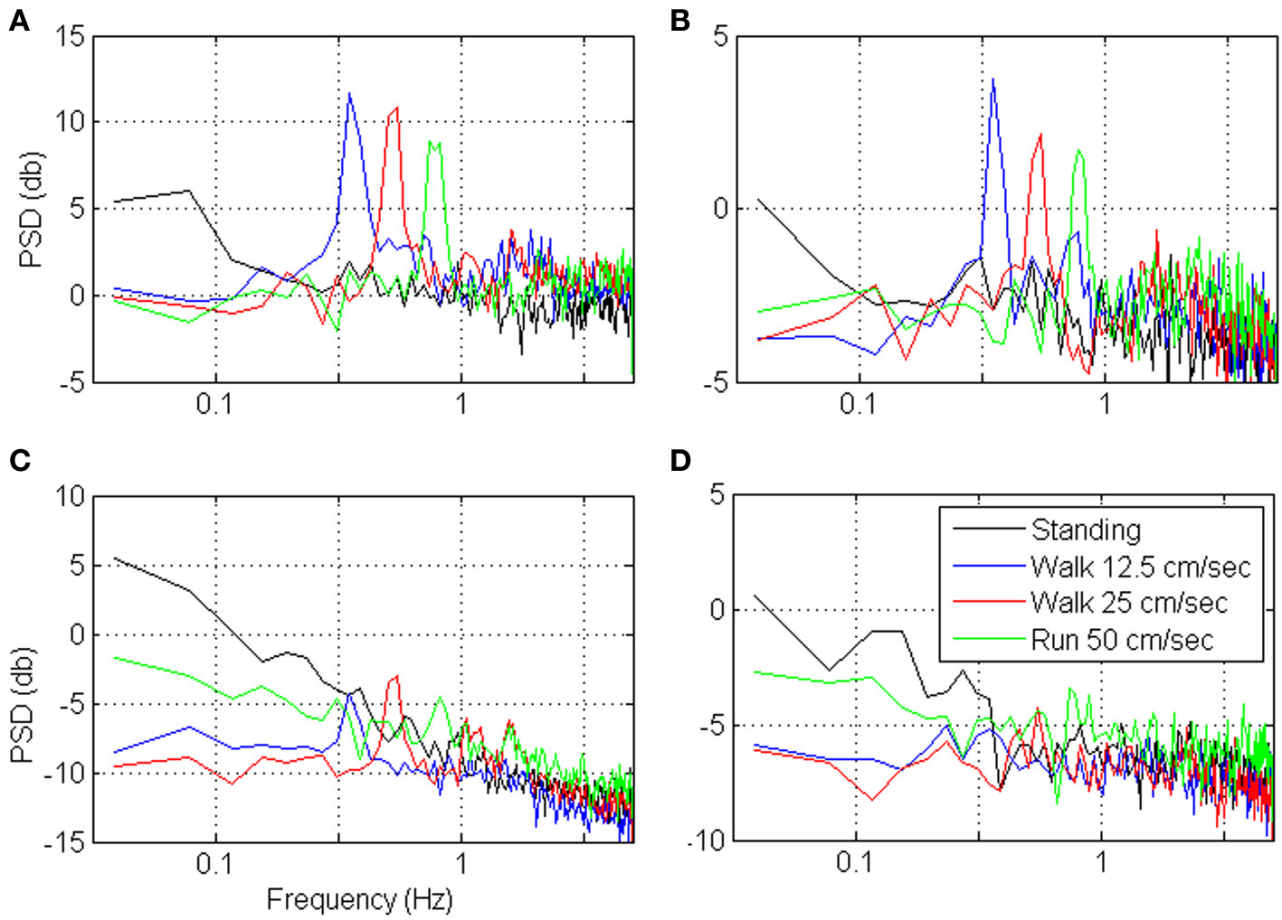
**Power Spectrum Density (PSD) of the neural activity analyzed in Figure 9**. PSDs were computed from the binned neural activity with 100 ms bins and frequency resolution of 0.039 Hz.

Figures 9, 10 depict the estimated *S*$\hat{N}$*R*_*N*_*T*__/*T* and the spectrum of representative cortical units in the motor leg area while the monkey was standing (black) or walking at increasing speeds (12.5, 25, and 50 cm/s). The *S*$\hat{N}$*R*_*N*_*T*__/*T* curves in Figures 9A,B peak at progressively shorter bin-widths as the speed of walking increases. During standing, the peak *S*$\hat{N}$*R*_*N*_*T*__ /*T* is shallower and appears at longer bin-widths than during walking. Figures 10A,B indicate that the spectrums of the spike-counts (in 100 ms bins) of these spike-trains are dominated by single spectral lines that appear at higher frequencies as the speed of walking increases. During standing, the dominating spectral line appears at very low frequencies and is smaller compared to the spectral lines during walking. Thus, this simple case, in which the spectrums are dominated by single spectral lines, helps to demonstrate the expected effect of changes in the spectral content of the modulating signals on the timescale. However, the timescale emerges from the interaction between the spectral content and the refractory properties of the individual unit. Hence, even though the dominating spectral lines appear in the same frequencies (Figures 10A,B) the timescales are unit specific (Figures 9A,B).

In contrast, the neural activity from other units in the leg area are characterized by *S*$\hat{N}$*R*_*N*_*T*__/*T* curves that either decrease without peaking (Figure 9D) or depict a peak that does not shift as expected with the speed of walking (Figure 9C). In both cases the neural activity during standing is characterized by higher *S*$\hat{N}$*R*_*N*_*T*__/*T* than during walking. The corresponding spectrums (Figures 10C,D) indicate that the power of the spike-counts recorded from these two units is indeed larger during standing than during walking. The spectral lines associated with the speed of walking (at the frequencies in which they appear in the spectrums depicted in panels A and B) are very small. Finally, for both units the neural activity recorded during running has more power in the low-frequency range than during walking. This may explain the unexpected shift in the peak of the *S*$\hat{N}$*R*_*T*_ /*T* toward longer rather than shorter bin-widths when the Monkey is running compared to walking (Figure 9C). The convex shape of the *S*$\hat{N}$*R*_*N*_*T*__/*T* curve in Figure 9D even at short bin-widths suggests that the activity of this unit is dominated by excitatory recovery effects, and the timescale is not conclusive. Nevertheless, since the *S*$\hat{N}$*R*_*N*_*T*__/*T* curves are similar during walking and running (independent of the speed) but much higher during standing, it can be concluded that signals associated with the frequency of walking have little effect on the spike-rate of these two units, and that their activity is more strongly modulated during standing.

Additional analysis (not shown) indicates that the mean *S*$\hat{N}$*R*_*N*_*T*__/*T* and mean normalized *S*$\hat{N}$*R*_*N*_*T*__/*T* across significantly modulated units with peak above 0.5 s^−1^ (*n* = 48) depict a wide peak around 50–110 ms, in most cases. The only exception is the normalized *S*$\hat{N}$*R*_*N*_*T*__/*T* during standing, which is highest at the shortest bin-width analyzed (25 ms). Around the peak, the magnitude of the *S*$\hat{N}$*R*_*N*_*T*__/*T* are similar independent of the speed so the curves overlap each other. At longer bin-widths *S*$\hat{N}$*R*_*N*_*T*__/*T* during slow walking is higher than during fast walking or running, and is highest during standing.

## Conclusions and discussion

### SNR estimation

We suggest a computational framework for estimating the *SNR* in spike trains without relying on any assumption about the encoded signals. The suggested approach captures all the encoded signals, including hidden and internal signals, and not only those controlled experimentally. The proposed approach has two limitations: (i) it accounts also for possible task irrelevant rate modulations, and (ii) the estimation method is derived under the assumption that the spike-trains are realization of DSPP. The first limitation is a necessary side-effect of capturing the hidden or internal signals. The second limitation is partially relaxed by considering dead-time modified DSPP and doubly stochastic Gamma processes.

The dependency of RM-*SNR* on bin-width was first analyzed for DSPP and two asymptotes were derived for bin-widths that are either very short or very long compared with the inverse of the bandwidth. In particular, it was shown that RM-SNR increases linearity at short bin-widths and reaches saturation at long bin-widths. Extending the analysis to dead-time modified DSPP, it was shown that the estimated RM-*SNR* is reduced by both scaling and shifting factors that do not depend on the bin-width. The bias effect becomes more significant as the RM-*SNR* becomes smaller at shorter bin-widths. Using simulations of doubly-stochastic Gamma processes, we also evaluate other recovery effects in which the occurrence of a spike temporarily and partially inhibits (as in relative refractory period) or enhances the probability of firing.

### Inhomogeneous vs. doubly stochastic point processes

Doubly stochastic point processes are inhomogeneous point processes with stochastic rate function. The alternative model for describing rate modulations is the inhomogeneous point process with a deterministic rate function (Cox and Isham, 1980; Kass and Ventura, 2001; Nawrot et al., 2008). The deterministic model is based on the assumption that the rate function depends solely on the experimentally controlled conditions, and thus that it can be estimated from the post-stimulus time histogram. This assumption might be valid for peripheral neurons that are affected almost exclusively by the experimentally controlled stimulus, but less for cortical neurons which may encode additional hidden signals, not directly under experimental control. These signals may vary from trial-to-trial and thus can only be modeled as stochastic. During movements, motor planning (Churchland et al., 2006a,b) and prediction errors (Wolpert and Ghahramani, 2000; Shadmehr and Krakauer, 2008), for example, may vary from trial-to-trial, while in decision making the accumulated evidence (while potentially sharing a common trend), may vary from trial-to-trial (Churchland et al., 2011). In these cases, doubly stochastic point processes are essential for capturing the variance of all the signals that might be encoded in the neural activity.

### Variance decomposition

Under the assumption that spike-trains are realizations of DSPPs, the variance of the spike-counts is decomposed into two terms associated with the signal and noise. A similar decomposition was developed in Churchland et al. (2011) where the resulting two terms were described as the variance of the underlying “intensity command” and the variance of the neural activity given that command. That decomposition was applied to uncover how trial-to-trial neural variability changes during decision making and reveal the underlying nature of neural processing. While the application and some of the analysis details differ (as discussed below), we share the most important hypotheses: (i) the neural activity may represent hidden signals, i.e., signals that are not directly controlled and hence may vary from trial to trial, (ii) decomposition of the variance of neural activity facilitates estimating the variance of the underlying rate, including modulations by those hidden signals, and (iii) variations in the variance of the underlying rate with time (or task) may reveal important information about the neural processing.

The decomposition developed in Churchland et al. (2011) is based on the total variance equation, which holds for any doubly stochastic point process. The total variance is decomposed into the variance of the conditional expectation (the variance of the expected spike-count given the rate parameter and the point process variance (the expectation of the conditional variance). Under the restriction to DSPP made here, the first term equals the variance of the rate parameter, which is assumed to encode the modulating signals. Otherwise, the relationship of the first term to the variance of the rate parameter is more complex. In particular, for dead-time modified DSPPs and for DSGPs, the first term is proportional to the variance of the rate-parameter but not equal to it (see Equations B1 and B6 for the dead-time modified DSPP). Hence, the restriction to DSPP made here assures that the meaning of the first term is clearly defined. Furthermore, it enables explicit analysis of the effect of the refractory period on both terms. Another major difference is that the analysis in Churchland et al. (2011) was applied to estimate the synchronized trial-to-trial variance in the underlying rate-process (intensity command) at each time along the trial. In contrast, we applied the analysis to estimate asynchronous variance in the underlying rate process.

### Timescales of neural activity

As bin-width increases, *SNR* increases but update-rate is compromised (Wu et al., 2006). This trade-off is captured by the ratio of *SNR* to bin-width, *SNR*_*T*_/*T*. Thus, the bin-width at which *SNR*_*T*_/*T* peaks optimizes the trade-off between *SNR* and update rate and is suggested as a criterion for characterizing the timescales of neural rate-coding.

Our analysis suggests that the estimated *S*$\hat{N}$*R*_*N*_*T*__/*T* is affected by both the spectral content of the encoded signals and the recovery properties of the unit. The effects of the bandwidth and absolute refractory period were analyzed theoretically and demonstrated via simulations of DSPPs and dead-time modified DSPPs. Their conflicting effects give rise to a peak in the *S*$\hat{N}$*R*_*N*_*T*__/*T* curve, which characterizes the timescale of neural rate-coding.

### Deviations from the DSPP assumption

The DSPP assumption is violated when the firing rate depends on the history of the spike train. In these cases, the estimated *S*$\hat{N}$*R*_*N*_*T*__ may differ from the actual *SNR*_*T*_, due to history-dependent modulations of the instantaneous rate. We restrict the analysis to the simple, yet common case, of renewal point processes, in which the dependency on the history is captured by the time elapsed since the last spike. The effect of absolute refractory period was analyzed theoretically, and was shown to result in a peak in the *S*$\hat{N}$*R*_*N*_*T*__/*T* curve. Thus, the dead-time limits the benefit of reducing the bin-width, since the estimated *S*$\hat{N}$*R*_*N*_*T*__ becomes increasingly distorted. The bin-width at which the peak occurs maximizes the trade-off between *S*$\hat{N}$*R*_*N*_*T*__ and update rate, and characterizes the timescale of neural rate-coding.

Using simulations we also demonstrated that relative refractory periods have similar effect, resulting in *S*$\hat{N}$*R*_*N*_*T*__/*T* curves that are also characterized by a peak. So the timescales are similarly characterized by the bin-widths at which the *S*$\hat{N}$*R*_*N*_*T*__/*T* curves peak. In contrast, when the recovery period is characterized by enhanced, rather than inhibited, probability of firing, the *S*$\hat{N}$*R*_*N*_*T*__/*T* over-estimates (rather than under-estimates) the *SNR*_*T*_/*T*. Thus, as the bin-width decreases, *S*$\hat{N}$*R*_*N*_*T*__/*T* increases without peaking or saturating. In those cases, the appropriate time-scale is not conclusive from the proposed analysis, since the higher *S*$\hat{N}$*R*_*N*_*T*__/*T* at short bin-widths reflect recovery effects rather than actual signal modulations. Further analysis is needed to select the bin-width that optimizes the trade-off between high *S*$\hat{N}$*R*_*N*_*T*__/*T* and small distortion due to the excitatory recovery effect.

### Timescales during BMI experiments

Estimating RM-*SNR* from neural activity recorded during BMI experiments, we observe that while its magnitude depends on the BMI mode and on training, the timescale revealed by the peak of the *S*$\hat{N}$*R*_*N*_*T*__/*T* curve remains the same.

The observation that RM-*SNR* increases when switching to brain control indicates that the variance of the rate increases—and thus that the variance of some of the encoded signals increases. The observation that the timescales remain invariant suggests that those signals are task-relevant, and we are currently investigating the hypothesis that they include state estimation and control signals, within the framework of optimal control. However, their exact nature and decoding remains an open issue.

Most of the analyzed *S*$\hat{N}$*R*_*N*_*T*__/*T* curves (62% on average across the different control modes, *n* = 109) were characterized by a wide peak at 50–150 ms. For this class of curves (and for the 15% of curves that peak at longer bin-widths), decreasing the bin-width below the peak may compromise decoding, unless the effect of the refractory period is accounted for. Indeed BMI applications based on spike-counts used bin-widths in the range between 30 and 100 ms (Wessberg et al., 2000; Serruya et al., 2002; Kim et al., 2008; Velliste et al., 2008), consistent with the range of timescales revealed here. In particular, the BMI experiments analyzed here were conducted with 100 ms bins (Carmena et al., 2003) within the wide peak of the estimated *S*$\hat{N}$*R*_*N*_*T*__/*T* curve depicted in Figure 4B. Our analysis suggests that this selection provides a good trade-off between the *SNR* and update rate.

Curves whose peak was below 50 ms were divided into those that were convex or concave at short bin-widths (15 and 8% on average, respectively). Concave shape at short bin-widths is expected from *S*$\hat{N}$*R*_*N*_*T*__/*T* curves derived from DSPPs, where this ratio should saturate at short bin-widths as detailed in Section Timescales Analysis (and Figure 2A). In this case, the range of bin-widths at which the curve saturates is indicative of the relevant timescales for neural decoding. At longer bin-widths, the slower than linear increase in *SNR* does not justify the reduction in update rate. Convex *S*$\hat{N}$*R*_*N*_*T*__/*T* are expected when the unit has excitatory recovery period (in which the probability of firing is enhanced). As detailed above, the timescale analysis in this case is not conclusive.

Since the relevant timescales vary across individual units, it might be beneficial to apply different bin-widths when binning different units. This may explain the potential advantage of multi-resolution binning (Kim et al., 2005). Recent decoding methods are based on point process filters that operate at 5 ms bins (Shanechi et al., 2013a,b). The BMI tested in Shanechi et al. (2013a) was based on multi-unit recording, where the dead-time effect might be negligible, and hence the timescales could be short. Furthermore, point process filters are based on estimating the conditional probability of the spike-counts given the rate, which is computationally more efficient when the bin is small (and the number of spikes that may occur with significant probability is limited). Hence, the choice of small bins may reflect computational constraints. The analysis conducted here suggests that unless recovery effects are negligible or accounted for, very short bin-widths may compromise the trade-off between *SNR* and update-rate.

### Timescales during locomotion experiments

The overall shape of *S*$\hat{N}$*R*_*N*_*T*__/*T* curves estimated from spike trains recorded during walking is similar to those derived from the spike trains recorded during the BMI experiments. However, the peak location in the locomotion experiments varied with the task, at least for some units, and shifted to lower bin-widths when the speed of walking increased. This is in agreement with the expected change in the bandwidth of encoded signals associated with the experimentally controlled speed of walking. Indeed, units whose timescales vary in this way are characterized by PSDs dominated by a single peak at the frequency of walking. Thus, the locomotion test-case demonstrates the expected effect of changes in the spectral content of the modulating signals on the timescales. However, timescales depend not only on the spectral content but also on the refractory properties of the units. So even if the spectral content is similar the timescales are unit-specific.

In summary, we developed a method for assessing the *SNR* and timescales of neuronal activity independent of the encoded signals and observe that many units depict well-defined timescales of the order of tens to hundreds msec. The timescale emerges from the interaction of the recovery properties of the unit and the spectral content of the modulating signals. In particular, the variations of the estimated *S*$\hat{N}$*R*_*N*_*T*__/*T* at short bin-widths is related to the recovery properties of the unit. During a BMI experiment, the average timescale across the population agrees well with the experimentally selected bin-width, though individual units depict different timescales. Furthermore, while *SNR* increases after switching to brain control and decreases with training, timescales remain similar. During locomotion, the analysis identified units whose timescales varied consistently with the experimentally controlled speed of walking, though the specific timescale reflected also the recovery properties of the unit. Hence, the proposed method provides a hypothesis free tool for investigating possible contributions of hidden signals, not under experimental control, to neural modulations and the effect of task conditions on their magnitude and timescales.

### Conflict of interest statement

The authors declare that the research was conducted in the absence of any commercial or financial relationships that could be construed as a potential conflict of interest.

## Appendix A: bandwidth effect

The binning process is equivalent to passing the instantaneous spike-rate through a finite time integrator followed by down-sampling and spike-counting (see Bair et al., 1994 for a similar formalization of the recording of spike-trains with finite time resolution). Specifically, the integrated spike-rate Λ_*T*_ (*t*) = λ_0_ *T* + $\tilde{\Lambda }$_*T*_ (*t*), where ${\tilde{\Lambda }}_{T}(t)=\int_{t-T/2}^{t+T/2}\tilde{\lambda }(\sigma )d\sigma$, is obtained by convolving the time-dependent component of the instantaneous spike-rate $\tilde{\lambda }$ (σ) with a rectangular window of duration *T*: $\tilde{\Lambda }$_*T*_ (*t*) = $\tilde{\lambda }$ (*t*) ⊗ *W*_*T*_ (*t*) [⊗ denotes convolution, and *W*_*T*_ (*t*) is a rectangle signal of duration *T* (Bair et al., 1994)]. The effect of down-sampling will be considered later. For now we note that the spectrum of $\tilde{\Lambda }$_*T*_ (*t*), denoted by *S*_$\tilde{\Lambda }$_ (ω), is related to the spectrum *S*_$\tilde{\lambda }$_ (ω) of $\tilde{\lambda }$ (*t*) by: *S*_$\tilde{\Lambda }$_ (ω) = |*G*_*W*_*T*__ (ω)|^2^ *S*_$\tilde{\lambda }$_ (ω), where $\left|G_{W_{T}}(\omega )\right|=T\left(\frac{\sin(\omega T/2)}{\omega T/2}\right)$ is the Fourier transform of the rectangular window (Bendat and Peirsol, 2000). The variance of Λ_*T*_ (*t*) can be computed from the spectrum *S*_$\tilde{\Lambda }$_ (ω) as: $Var[\Lambda_{T}(t)]=\frac{1}{2\pi }\int_{-\infty }^{\infty }S_{\tilde{\Lambda }}(\omega )d\omega$, while its mean is *E*[Λ_*T*_ (*t*)] = E[N_*T*_ (*t*)] = λ_0_ *T*. Using Equation (3), the *SNR* of the binned spike-counts is given by:

(A1) $$SNR_{T}=\frac{1}{\lambda_{0}T}\frac{1}{2\pi }\int_{-\infty }^{\infty }{\left|G_{W_{T}}(\omega )\right|}^{2}S_{\tilde{\lambda }}(\omega )d\omega$$

Assuming that the spectrum of the instantaneous spike-rate *S*_$\tilde{\lambda }$_ (ω) is band limited in the range [−ω_max_, ω_max_], the *SNR* reaches two asymptotes for short and long bin-widths. For short bin-widths *T*, i.e., when ω_max_ «2π /*T*, the transfer function of the rectangular window |*G*_*W*_*T*__ (ω)| in the range [−ω_max_, ω_max_] can be approximated by |*G*_*W*_*T*__ (0)| = *T*, so, from Equation (A1):

(A2) $$SNR_{T}≅\frac{1}{\lambda_{0}T}\frac{T^{2}}{2\pi }\int_{-\omega_{\max}}^{\omega_{\max}}S_{\tilde{\lambda }}(\omega )d\omega =\frac{P_{\tilde{\lambda }}}{\lambda_{0}}T,$$

where $P_{\tilde{\lambda }}=\frac{1}{2\pi }\int_{-\omega_{\max}}^{\omega_{\max}}S_{\tilde{\lambda }}(\omega )d\omega$ is the power of the time-dependent component of the instantaneous rate. Hence, at short bin-widths, the *SNR* of spike-counts generated from DSPP with band-limited instantaneous rate should increase linearly with the bin-width *T*.

For long bin-widths *T*, i.e., ω_max_ »2π /*T*, the Fourier transform of the rectangular window is concentrated around zero, so only low frequencies pass the averaging process and affect the average rate. Denoting by *S*_0_ the spectrum of the instantaneous rate at low frequencies, Equation (A1) implies that at long bin-widths the *SNR* saturates at:

(A3) $$SNR_{T}\underset{T\to \infty }{\to }\frac{1}{\lambda_{0}T}\frac{S_{0}}{2\pi }\int_{-\infty }^{\infty }{\left|G_{W_{T}}(\omega )\right|}^{2}d\omega =\frac{S_{0}T}{\lambda_{0}T}=\frac{S_{0}}{\lambda_{0}}.$$

Binning is usually performed in non-overlapping windows, so the binned spike-counts are related to the samples of the integrated spike-rate taken at sampling interval *T*. At short bin-widths, the Nyquist frequency ω_*Ny*_ ≡ ω_*sampling*_ /2 ≡ π /*T* satisfies the Nyquist criterion ω_*Ny*_ >ω_max_. Hence, there is no aliasing, and Equation (A2) holds, implying that the *SNR* of binned spike counts should increase linearly with the bin-width. At long bin-widths, the Nyquist criterion is not satisfied, and the side bands of the spectrum of the rectangular window |*G*_*W*_*T*__ (ω)|^2^ fold back into the main lobe. Nevertheless, as long as the spectrum is constant over most of the side-lobes, the total power in the main lobe (including all the folded back side-lobes) is the same as the integral computed in Equation (A3) (Bair et al., 1994). Hence the *SNR* of binned spike counts should saturates at long bin-widths.

## Appendix B: SNR of dead-time modified DSPP

Assuming the dead-time is short compared to the dynamics of the instantaneous rate, the statistics of the spike-counts depend on the dead-time modified integrated rate: ${\bar{\Lambda }}_{T}=\int_{t-T/2}^{t+T/2}\frac{\lambda (\sigma )}{1+\tau_{d}\lambda (\sigma )}d\sigma$, and are given by [see Equations 3 and 9 in Vannucci and Teich (1981)]:

(B1) $$E[N_{T}]=E\left[{\bar{\Lambda }}_{T}\right]$$

(B2) $$E[N_{T}^{2}]=E\left[{\left({\bar{\Lambda }}_{T}\right)}^{2}\right]+E\left[\int_{t-T/2}^{t+T/2}\frac{\lambda (\sigma )}{{\left[1+\tau_{d}\lambda (\sigma )\right]}^{3}}d\sigma \right]$$

So:

(B3) $$Var[N_{T}]=Var[{\bar{\Lambda }}_{T}]+E\left[\int_{t-T/2}^{t+T/2}\frac{\lambda (\sigma )}{{\left[1+\tau_{d}\lambda (\sigma )\right]}^{3}}d\sigma \right]$$

Substituting Equations (B1) and (B3) in Equation (4), the estimated *SNR* from a dead-time modified DSPP is:

(B4) $$S\hat{N}R_{N_{T}}(\tau_{d})=\frac{Var\left[{\bar{\Lambda }}_{T}\right]+E\left[\int_{t-T/2}^{t+T/2}\frac{\lambda (\sigma )}{{\left[1+\tau_{d}\lambda (\sigma )\right]}^{3}}d\sigma \right]-E\left[{\bar{\Lambda }}_{T}\right]}{E\left[{\bar{\Lambda }}_{T}\right]}$$

The *SNR* associated with the dead-time modified integrated rate, *S**N**R*_*T*_, can be defined in a similar way to the definition of *SNR*_*T*_ for the original integrated rate (Equation 3):

(B5) $$S\bar{N}R_{T}\equiv \frac{Var\left[{\bar{\Lambda }}_{T}\right]}{E\left[{\bar{\Lambda }}_{T}\right]}$$

For τ_*d*_ $\tilde{\lambda }$ (σ) « 1, the integral defining Λ_*T*_ can be approximated by: ${\bar{\Lambda }}_{T}≅\frac{1}{1+\tau_{d}\lambda_{0}}\int_{t-T/2}^{t+T/2}\lambda (\sigma )d\sigma =\frac{\Lambda_{T}}{1+\tau_{d}\lambda_{0}}$ so its statistics can be approximated by:

(B6) $$\;E[{\bar{\Lambda }}_{T}]≅\frac{E\left[\Lambda_{T}\right]}{1+\tau_{d}\lambda_{0}}$$

(B7) $$Var[{\bar{\Lambda }}_{T}]≅\frac{Var\left[\Lambda_{T}\right]}{{\left[1+\tau_{d}\lambda_{0}\right]}^{2}}$$

Hence, the *S**N**R*_*T*_ can be related to *SNR*_*T*_ by:

(B8) $$S\bar{N}R_{T}(\tau_{d})≅\frac{SNR_{T}}{\left[1+\tau_{d}\lambda_{0}\right]}$$

Using a similar approximation, the second term in Equations (B2–B4) can be approximated as:

(B9) $$E\left[\int_{t-T/2}^{t+T/2}\frac{\lambda (\sigma )}{{\left[1+\tau_{d}\lambda (\sigma )\right]}^{3}}d\sigma \right]≅\frac{E\left[\Lambda_{T}\right]}{{\left[1+\tau_{d}\lambda_{0}\right]}^{3}}$$

Substituting these approximations in Equation (B4) results in an approximated relationship between the estimated *SNR* from a dead-time modified DSPP and the *SNR* of a dead-time free DSPP having the same instantaneous rate:

(B10) $$\begin{array}{l} S\hat{N}R_{N_{T}}(\tau_{d})≅S\bar{N}R_{T}-\frac{\tau_{d}\lambda_{0}\left(2+\tau_{d}\lambda_{0}\right)}{{\left[1+\tau_{d}\lambda_{0}\right]}^{2}}≅\frac{SNR_{T}}{\left[1+\tau_{d}\lambda_{0}\right]}\\ \;-\frac{\tau_{d}\lambda_{0}\left(2+\tau_{d}\lambda_{0}\right)}{{\left[1+\tau_{d_{0}}\right]}^{2}} \end{array}$$

Note that the second term increases with the dead-time both absolutely and as a fraction of the first term.
